# Sociodemographic and clinical predictors of quality-of-life outcome in children and young people with primary brain tumour in Karachi, Pakistan: a prospective cohort study

**DOI:** 10.1136/bmjpo-2024-002505

**Published:** 2024-12-11

**Authors:** Nida Zahid, Syed Ather Enam, Thomas Mårtensson, Iqbal Azam, Naureen Mushtaq, Mariya Moochhala, Aneesa Hassan, Faiza Kausar, Saqib Bakhshi, Lal Rehman, Farrukh Javeed, Muhammad Nouman Mughal, Sadaf Altaf, Salman Kirmani, Nick Brown

**Affiliations:** 1 Department of Surgery, The Aga Khan University, Karachi, Pakistan; 2 Global Health and Migration Unit, Department of Women’s and Children’s Health, Uppsala Universitet, Uppsala, Sweden; 3 Department of Community Health Sciences, The Aga Khan University, Karachi, Pakistan; 4 Department of Oncology, The Aga Khan University, Karachi, Pakistan; 5 Department of Psychiatry, The Aga Khan University Hospital, Karachi, Pakistan; 6 Department of Neurosurgery, Jinnah Postgraduate Medical Centre, Karachi, Pakistan; 7 Division of Women & Child Health, The Aga Khan University, Karachi, Pakistan; 8 Department of Paediatrics, The Aga Khan University, Karachi, Pakistan

**Keywords:** Epidemiology, Neurosurgery, Psychology, Child Psychiatry, Adolescent Health

## Abstract

**Background:**

Children and young people (CYP) with primary brain tumour (PBT) are at high risk for developing late effects, potentially affecting long-term quality of life (QoL). In low-income and middle-income countries, QoL has not been studied in depth in CYP. In the present study, CYP treated for PBTs in Pakistan were evaluated regarding (A) mean change in QoL scores pretreatment and 12 months post-treatment and (B) predictors of change in QoL scores 12 months post-treatment.

**Methods:**

A prospective cohort study was conducted from November 2020 to July 2023. CYP aged 5–21 years, with newly diagnosed PBTs, were recruited from tertiary care centres in Karachi, Pakistan. QoL was assessed using the Paediatric Quality of Life Inventory 4 generic and brain tumour module, pretreatment and at 12 months post-treatment, by a trained psychologist.

**Results:**

A total of 48 patients diagnosed with PBTs were enrolled in the study. At the 12-month post-treatment, 25 (52%) of the patients were reassessed, while 23 (48%) were lost to follow-up. There was no significant difference in mean global QoL scores of patients at 12 months post-treatment. On multivariable analysis, there was a statistically significant improvement in mean global QoL scores among those who underwent total surgical tumour resection (beta 7.7; 95% CI 0.9, 14.5) and maximum safe surgical tumour resection (beta 10.6; 95% CI 4.7, 16.6). However, there was a significant decline in mean global QoL scores among those who had hydrocephalous at diagnosis managed with a shunt and/or external ventricular drain (EVD) (beta −10.0; 95% CI −14.5, –5.5).

**Conclusion:**

This study found a decline in mean global QoL scores among those with hydrocephalous at diagnosis who were managed with a shunt and/or EVD but an improvement in those who underwent total or maximum safe surgical tumour resection. Larger-scale studies are needed to comprehensively evaluate and validate these outcomes.

**WHAT IS ALREADY KNOWN ON THIS TOPIC**

- Cross-sectional studies from high-income countries have assessed quality-of-life (QoL) outcomes in children and young people with primary brain tumours; however, longitudinal studies are scarce. Similarly, in-depth studies on QoL predictors in this patient group are lacking in low-income and middle-income countries (LMICs). In Pakistan, only one cross-sectional study has examined QoL outcomes in children with craniopharyngioma.

**WHAT THIS STUDY ADDS**

- This longitudinal study addresses this research gap by identifying factors that may impact QoL outcomes for children and young people with primary brain tumours in LMICs.

**HOW THIS STUDY MIGHT AFFECT RESEARCH, PRACTICE OR POLICY**

- This study provides data on factors that may predict QoL outcomes in children and young people with primary brain tumours, specifically from an LMIC perspective. These findings suggest the need for targeted early psychological screening for higher-risk groups. To enhance generalisability, future research should prioritise larger-scale, multicountry studies.

## Introduction

 Primary brain tumours (PBTs) rank as the second most common childhood malignancies worldwide.^1^ The estimated 5-year survival rate for children and young people (CYP) has significantly improved, increasing from 55% in 1975 to 76% in 2017.^2^ This progress has led to a rise in adverse late effects.^3 4^ These late effects can significantly impact the quality of life (QoL) and overall well-being of survivors.^5^

Although QoL has been evaluated in CYP with PBTs in high-income countries,^6^ the majority of these studies are cross-sectional. Longitudinal studies are limited and often lack clearly defined baselines for comparison.^6^ Furthermore, research from low and middle-income countries (LMICs) is scarce, with only three cross-sectional studies reporting QoL outcomes in children with PBTs.^7^,^9^ No in-depth longitudinal studies have explored QoL outcomes in this population within LMICs.

It is important to recognise that sociocultural, psychosocial and biological factors can vary significantly between and within LMICs and high-income countries eg, Organization for Economic Co-operation and Development (OECD) member countries. Therefore, extrapolating findings from high-income settings to LMICs may not be appropriate.

This prospective longitudinal study conducted at two tertiary care hospitals in Karachi, Pakistan aims to (A) assess the change in QoL scores in CYP with PBTs, pretreatment and 12 months post-treatment and (B) evaluate the potential impact of sociodemographic, tumour and treatment factors on changes in global QoL scores.

## Materials and methods

### Study design and setting

A prospective cohort study was conducted at a private tertiary care hospital in Karachi, Pakistan: Aga Khan University Hospital (AKUH) and Jinnah Postgraduate Medical Centre (JPMC).

### Study population and eligibility criteria

#### Inclusion Criteria

Patients aged 5–21 years residing in Pakistan were eligible if they had a newly diagnosed PBT, either benign or malignant, of any grade, and had not received prior treatment for a brain tumour. PBTs were defined as ‘tumours that originate from the tissues of the brain or the brain’s immediate surroundings’.^10^ Diagnoses were based on the 2021 WHO Classification of Tumours of the Central Nervous System.^11^ This was confirmed by MRI.

#### Exclusion criteria

Patients with a history of psychiatric disorders (eg, schizophrenia, depression).

Patients with neurological conditions (eg, attention-deficit/hyperactivity disorder, autism).

Patients with physical comorbidities (eg, severe vision or hearing impairment, chronic obstructive pulmonary disease, other types of cancer).

Patients with language barriers.

Patients with lack of formal education.

Patients with critical condition.

Patients declining to participate.

#### Sample size

The sample size was calculated to detect a clinically meaningful change in QoL scores.^12^ A minimum sample of 48 patients with PBTs was required to achieve 80% power at a two-sided 5% significance level. This calculation accounted for a potential 65% attrition or non-response rate. The study aimed to detect at least a 10% change in mean QoL scores with a 20% or less change in the coefficient of variation, based on findings reported by Quast *et al* and Penn *et al* from Philadelphia, USA and Bristol, UK, respectively.^13 14^

#### Data collection procedure

Participants were recruited using a non-probability purposive sampling method. A trained research assistant approached potentially eligible patients with newly diagnosed PBTs at their scheduled appointments in the surgical and oncology clinics of AKUH and JPMC. 48 patients were enrolled in the study, with 29 from AKUH and 19 from JPMC.

#### Independent variables

Information on the following independent variables was obtained: (1) demographics; (2) tumour factors that included tumour histopathology, tumour location (supratentorial, infratentorial, suprasellar and sellar), history of pretreatment seizures, the presence or absence of hydrocephalus at diagnosis confirmed radiologically by MRI and (3) treatment-related factors that included type of treatment (surgical resection, chemotherapy, radiotherapy and biopsy), type of surgical resection of tumour, that is, total (100% removal of tumour), maximum safe (>90% removal of tumour while prioritising safety) and subtotal (<90% removal of tumour). These definitions are based on institutionally established cutoffs, determined through post-MRI assessments of residual tumour. An external ventricular drain (EVD) or ventriculoperitoneal shunt (VPS) is used for patients with hydrocephalus at diagnosis.

Neurocognitive outcomes (verbal and non-verbal) were assessed by a psychologist at both the study sites pretreatment and at 12 months post-treatment by using validated assessment instruments. Verbal intelligence was measured by the Slosson Intelligence tool.^15^ Perceptual reasoning was assessed by Raven’s Progressive Matrices^16 17^ and processing speed was assessed using the Wechsler Intelligence Scale for Children and Wechsler Adult Intelligence Scale.^18^,^20^

#### Dependent variable

##### Quality of life

QoL was assessed at baseline (pretreatment) and 12 months post-treatment by a psychologist using the Paediatric Quality of Life Inventory (PedsQL), an internationally validated tool.^21^ The PedsQL 4 generic tool yields a total/global score and four subscale scores: physical, emotional, social and school functioning. Higher scores represent a better QoL. It comprises of child’s self-report form and parent’s proxy form for different age groups from 2 to 25 years.^22^ This study used the version translated and validated in Urdu.^23^

In addition to the generic scales, the PedsQL Brain Tumour Module was administered. The module comprises of six different scales: cognitive problems (seven items), pain and hurt (three items), movement and balance (three items), procedural anxiety (three items), nausea (five items),and worry (three items). Higher score indicates better health-related QoL, indicating fewer symptoms or problems.^24^ As this module was not previously validated in Urdu, it underwent translation and validation before the use in this study. The Content Validation Index (CVI) for relevance and clarity demonstrated excellent agreement for both child/young adult self-report (ages 5–21 years; CVI range: 0.82–0.93) and parent proxy-report (CVI range: 0.89–0.92).

To ensure confidentiality, assessments of participants and parents were conducted in a private room. The psychologist developed a treatment plan for any individual identified as having a significant decline in QoL scores at the 12-month post-treatment assessment.

### Statistical analysis

Data were analysed by using Stata V.12 (StataCorp). The QoL scores and other quantitative independent variables were reported as mean and SD or median IQR. The mean difference in QoL scores was assessed by paired t-test. Categorical variables were reported as frequency and percentages and assessed by χ^2^ or Fisher’s exact test. Univariate and multivariable analyses were performed using generalised estimating equations to determine the association of various independent factors with the 12-month change in global QoL scores. These factors included child demographics, parental factors (educational status, socioeconomic status), tumour-related and treatment-related factors, and neurocognitive outcomes. Models were adjusted for relevant covariates.

Unadjusted and adjusted beta coefficients with 95% CIs were calculated. All plausible interactions and confounders were assessed. Independent variables with a p<0.2 in the univariate analysis were considered significant for inclusion in the multivariable model, based on Bendel and Afifi,^25^ as using a more traditional significance level (<0.05) often fails to identify important predictors.^25^ However, a p<0.05 for multivariable analysis was considered statistically significant.

### Patient and public involvement

Patients or the public were not involved in the design, or conduct, or reporting, or dissemination plans of our research.

## Results

Of 60 newly diagnosed patients with PBTs screened for eligibility, 12 (20%) were excluded. Reasons for exclusion included language barrier (n=6), severe vision and/or hearing impairment (n=1), lack of formal education (n=2), critical condition requiring emergency care (n=2) and refusal to participate due to pain (n=1) (online supplemental file 1).

The remaining 48 patients were enrolled in the study, with 29 from AKUH and 19 from JPMC. 23 (48%) patients were lost to follow-up, of whom 10 (43%) died.

Participants and non-participants had similar demographic and baseline characteristics. However, a significantly higher proportion of CYP with PBTs who were loss to follow-up experienced post-treatment seizures and hydrocephalous managed with VPS or EVD (online supplemental file 2).

### Sociodemographic factors

The median age of participants at enrolment was 12.5 years (range: 9–16 years), and 29 (60%) were males. Sindh was the home province for 35 (73%) of the patients; the remaining 13 (27%) were from Punjab, Khyber Pakhtunkhwa and Gilgit Baltistan. Urdu was the mother tongue of 22 (46%) patients, while the remaining 26 (54%) spoke regional languages of Pakistan.

#### Parental sociodemographic factors

19 (40%) mothers and 26 (54%) fathers of the patients had attained an educational level of higher secondary school or above. In 38 (79%) households, fathers were the sole income earners. The median household monthly income was US$106 (IQR: US$71–US$283).

#### Tumour and treatment-related factors

17 (35%) patients were presented with infratentorial tumours, and 31 (65%) presented with supratentorial tumours. Based on tumour histopathology, 27 (56%) had low-grade tumours, and 13 (27%) had high-grade tumours, while the grade was unknown for 8 (17%). Pilocytic astrocytoma was the predominant subtype (12 patients, 25%), followed by medulloblastoma (7 patients, 15%), craniopharyngioma (5 patients, 10%), pituitary adenoma (5 patients, 10%), glioblastoma (4 patients, 8%), ependymoma (3 patients, 6%) and diffuse astrocytoma (1 patient, 2%). Three patients (6%) had other tumour types. The histopathology for eight patients (18%) was unknown as they did not undergo surgical intervention.

The median tumour size on MRI was 35 (IQR=9–90) cm^3^. 10 (21%) participants experienced preoperative seizures, and 23 (48%) had hydrocephalus at diagnosis. Eight (17%) patients had visual impairment. 23 (48%) patients were managed with a VPS and/or an EVD. Of these, 11 (48%) had shunt placement before surgery, and 12 (52%) had it after surgery. Four out of twenty five (16%) patients experienced postsurgery seizures.

26 (54%) patients underwent surgical intervention only, and 12 (25%) underwent a combination of surgery and adjuvant therapy (surgery and radiotherapy (n=3); surgery and chemotherapy (n=4) and surgery, chemotherapy, and radiotherapy (n=5)). One patient (2%) underwent radiotherapy only, while 9 (19%) had no intervention. Of those who underwent surgery, 25 (66%) had maximum safe resection, 10 (25%) underwent total resection, 2 (5%) underwent subtotal resection and 1 (3%) had only a biopsy.

Among the nine patients (age range: 11–21 years) who underwent radiotherapy, five (56%) received treatment using the CyberKnife laser modality, while information about the modality for the remaining three (33%) was unavailable. The number of radiotherapy sessions ranged from 2 to 34.

#### Pretreatment QoL scores of CYP (5–21 years) with PBTs with post-treatment 12 months follow-up versus loss to follow-up

Children with PBTs who were followed up at 12 months post-treatment compared with those lost to follow-up showed significantly higher QoL scores only in the following domains: global QoL, physical functioning, psychosocial functioning and worry (table 1).

**Table 1:** Comparison of pretreatment quality-of-life (QoL) scores of children and young people (CYP) (5–21 years) with primary brain tumour (PBT) followed at 12 months vs loss to follow-up

QoL domains	CYP with PBTs with 12 months follow-up	CYP with PBTs loss to follow-up	Mean difference in QoL
	**QoL scores Mean (SD)**		
**PedsQL core score**			
Global Score*	83.5 (7.8)	76.1 (13.2)	7.4 (0.9,13.9)
Physical health summary score(physical functioning)*	82.9 (14.0)	69.8 (22.2)	13.0 (2.1,24.0)
Psychosocial functioning*	85.5 (10.9)	76.6 (15.4)	3.8 (1.2,16.6)
Emotional functioning	76.6 (19.1)	70.9 (21.2)	5.7 (-5.9,17.4)
Social functioning	81.0 (8.0)	76.7 (15.4)	4.3 (-2.8,11.3)
School functioning*	82.8 (14.8)	66.7 (23.2)	16.1 (4.6,27.6)
**PedsQL brain tumour module score**			
Cognitive problems	79.9 (17.7)	72.7 (21.4)	7.1 (-4.2,18.5)
Pain and hurt	72.0 (24.7)	71.7 (23.6)	0.3 (-13.8,14.4)
Movement and balance	88.6 (16.4)	83.7 (25.6)	4.9 (-7.8.17.7)
Procedural anxiety	81.7 (27.1)	91.3 (19.5)	−9.6 (-23.3,4.0)
Nausea	81.2 (19.9)	85.0 (21.9)	−3.8 (-15,8.4)
Worry*	91.3 (18.9)	73.2 (31.4)	18.1 (2.8,33.5)

Higher scores indicate lower problem.

Global score is an average of QoL scores on physical, emotional, social and school functioning.Psychosocial functioning is an average of QoL scores on emotional, social and school functioning.

* Statistically significant at p<0.05 by independent t-test.

PedsQLPaediatric Quality of Life Inventory

#### Mean difference in QoL scores 12 months post-treatment

Social functioning scores increased at 12 months post-treatment (mean difference 14.8; 95% CI 9.0, 20.6). However, there was a significant decline in cognitive functioning at 12 months post-treatment

(mean difference −15.2; 95% CI −24.0, –6.4). No significant differences were observed in the remaining QoL domains at 12 months post-treatment (table 2).

**Table 2:** Mean difference in quality-of-life (QoL) scores 12 months post-treatment of children and young people (5–21 years) with primary brain tumour (n=25)

PedsQL domains	Mean pretreatment QoL scores (SD)	Mean post-treatment QoL scores (SD)	Mean difference (95% CI)
**PedsQL Core**			
Global score	82.9 (8.7)	82.0 (12.7)	−0.9 (−6.8, 4.9)
Physical functioning	81.0 (14.8)	79.0 (20.6)	−2.0 (−9.3, 5.3)
Psychosocial functioning	84.5 (11.2)	82.7 (11.4)	−1.7 (−8.7, 5.3)
Emotional functioning	74.6 (19.1)	69.4 (17.7)	−5.2 (−16.0, 5.6)
Social functioning*	80.2 (9.1)	95.0 (14.7)	14.8 (9.0, 20.6)
School functioning	83.0 (14.9)	83.8 (19.8)	0.8 (−8.5, 10.1)
**PedsQL brain tumour module**			
Cognition*	77.3 (20.7)	62.1 (21.1)	− 15.2 (−24.0, -6.4)
Pain and hurt	72.0 (24.8)	71.0 (21.5)	− 1.0 (−14.4, 12.4)
Movement	85.3 (21.8)	88.3 (24.8)	3.0 (−6.2, 12.2)
Procedural anxiety	81.7 (27.1)	76.0 (36.4)	− 5.7 (−19.7, 8.4)
Nausea	81.2 (20.0)	88.6 (20.3)	7.4 (−3.8, 18.7)
Worry	88.3 (23.0)	81.7 (29.1)	− 6.7 (−22.0, 8.7)

* Statistically significant at p<0.05 by using paired t-test.

PedsQLPaediatric Quality of Life Inventory

#### Regression analysis

On univariate analysis, several factors were associated with a significant decline in mean global QoL scores at 12 months post-treatment among CYP with PBTs. These factors included high-grade tumour, specific tumour histopathology (ependymoma and medulloblastomas), the presence of hydrocephalous at diagnosis managed with a shunt and/or EVD, low pretreatment processing speed scores, low post-treatment verbal intelligence scores and low household monthly income. Conversely, patients who underwent surgical tumour resection, including total and maximum safe resection, showed a significant increase in mean global QoL scores compared with those who did not undergo surgical intervention (table 3).

**Table 3:** Predictors of change in mean global quality of life scores of brain tumour patients of children and young people (5–21 years) with primary brain tumour (n=25)

Factors (n)	Unadjusted β coefficient (95% CI)	Adjusted β coefficient (95% CI)
Tumour histopathology		
Pilocytic astrocytoma (9)	(Reference)	
Ependymoma (1)	−13.6 (−24.7, –2.6)	
Craniopharyngioma (4)	−2.8 (−10.9, 5.4)	
Glioblastoma (3)	4.0 (−4.9, 12.9)	–
Medulloblastoma (1)	− 9.7 (−17.8, –1.6)	
Pituitary adenoma (2)	4.4 (−4.5, 13.3)	
Others (2)	− 0.1 (−9.0, 8.8)	
Grade of tumour		
Low grade (18)	(Reference)	
High grade (5)	− 5.5 (−11.8, 0.8)	–
Not known (2)		
Type of treatment		
No intervention (4)	(Reference)	
Surgery only (14)	8.9 (1.5, 16.3)	–
Combination of treatment (7)	6.7 (−1.6, 14.9)	
Type of surgical intervention†		
No intervention (4)	(Reference)	(Reference)
Total resection (7)	8.4 (0.6, 16.2)	7.7 (0.9, 14.5)*‡
Subtotal total resection (1)	− 3.7 (−15.9, 8.4)	−2.8 (−13.2, 7.6)
Maximum safe resection (13)	9.7 (2.9, 16.5)	10.6 (4.7, 16.6)*‡
Presence of hydrocephalous/shunt		
Yes (8)	−9.0 (−14.2, –3.9)	−10.0 (−14.5, –5.5)*‡
No (17)	(Reference)	(Reference)
Pretreatment processing speed scores (19)	0.2 (−0.1, 0.4)	–
Post-treatment verbal intelligence scores (24)	0.1 (−0.01, 0.2)	–
Household monthly income (USD)		
≤53 (4)	−10.8 (−18.7,–2.8)	–
53–159 (9)	-4.9 (−11.4, 1.6)	
159–318 (4)	-8.1 (−16.5, 0.3)	
318–3600 (8)	(Reference)	

* Combination of treatment: surgery, chemotherapy and radiotherapy.

† Type of surgery: total resection (100% of tumour removal); maximum safe resection (>90% of tumour removal); subtotal resection (<90% of tumour removal)*.*

‡ Significant at p<0.05 on multivariable analysis.

On multivariable analysis, there was a statistically significant improvement in mean global QoL scores (p<0.05) among those who underwent total surgical tumour resection (beta 7.7; 95% CI 0.9, 14.5) and maximum safe surgical tumour resection (beta 10.6; 95% CI 4.7, 16.6) compared with those with no surgical intervention. However, there was a statistically significant decline in mean global QoL scores among those with hydrocephalous at diagnosis managed with a shunt and/or EVD (beta −10.0; 95% CI −14.5, –5.5) (table 3).

### Discussion

The key finding in the present study was that patients undergoing total or maximum safe surgical tumour resection experienced significant improvement in their QoL scores at 12 months post-treatment. However, those with hydrocephalus at diagnosis, managed with a shunt and/or EVD, had a statistically significant decline in mean global QoL scores compared with those without hydrocephalus.

This study highlights the positive impact of surgical tumour resection on QoL, particularly total or maximum safe resection. These findings align with those reported by Musiol *et al* and Brown *et al* in paediatric PBTs^26 27^ and Kim *et al* in adults,^28^ where surgical intervention alleviated headaches and pain by reduction of mass effect, thereby improving the QoL. However, access to neurosurgical care remains a challenge in LMIC countries. In Pakistan, only 1 neurosurgeon per 139 300 people^29^ compared with a ratio of 1 per 80 000 in high-income OECD countries.^30^ Furthermore, limited resources, geographical disparities and financial burdens lead to delays in diagnosis, treatment and follow-up.^31^ In our study, 27% of patients resided in other provinces, patients from remote areas often face long journeys to access surgical facilities.^32^ Therefore, improving healthcare accessibility is crucial for early diagnosis, timely referral and prompt surgical intervention, ultimately enhancing the well-being and QoL of PBT patients.

We observed a significant decline in QoL outcomes among patients with hydrocephalus managed with a shunt and/or EVD. These findings are consistent with those reported by Penn *et al*^33^ and Bhat from the USA.^34^ Hydrocephalus can cause various neurological symptoms, including vision problems, balance and coordination issues, cognitive changes, difficulty concentrating and headaches, impacting daily activities.^33^ These patients may also struggle with peer relationships and academic achievement, negatively affecting their overall well-being.^35^ The symptoms associated with hydrocephalus can significantly impact QoL, emphasising the importance of early psychological screening and counselling for these patients.

Identifying factors that influence QoL can guide the development of targeted strategies to improve the overall well-being of children with PBTs. This requires ongoing collaboration among neurosurgeons, neuro-oncologists, psychologists, caregivers, rehabilitation centres and educational institutions.

This study’s strengths include its inclusion of a range of tumour phenotypes and complications, individual pretreatment and post-treatment psychological reviews, and the use of validated tools to assess QoL and neurocognitive outcomes. However, limitations include the relatively small sample size, which limited statistical power and generalisability, particularly due to loss to follow-up of 48% of the study participants at the 12-month re-assessment. The small number of patients within each histology phenotype precluded direct inferences for specific tumour types. Additionally, the possibility of selection bias cannot be disregarded, as patients undergoing surgery may inherently have better long-term prospects.

### Conclusions

This study found a decline in mean global QoL scores among those with hydrocephalous at diagnosis who were managed with a shunt and/or EVD but an improvement in those who underwent total or maximum safe tumour resection. Larger-scale studies are needed to comprehensively evaluate and validate these outcomes.

## supplementary material

10.1136/bmjpo-2024-002505 online supplemental file 1

10.1136/bmjpo-2024-002505 online supplemental file 2

## Data Availability

Data are available on reasonable request.
