# Supplementary figures and images for: Sociodemographic and clinical predictors of quality-of-life outcome in children and young people with primary brain tumour in Karachi, Pakistan: a prospective cohort study
Source: BMJ Paediatr Open. 2024 Dec 11;8(1):e002505. doi: 10.1136/bmjpo-2024-002505 (PMC11647362; doi:10.1136/bmjpo-2024-002505)

## Supplementary 1: Flow of the study

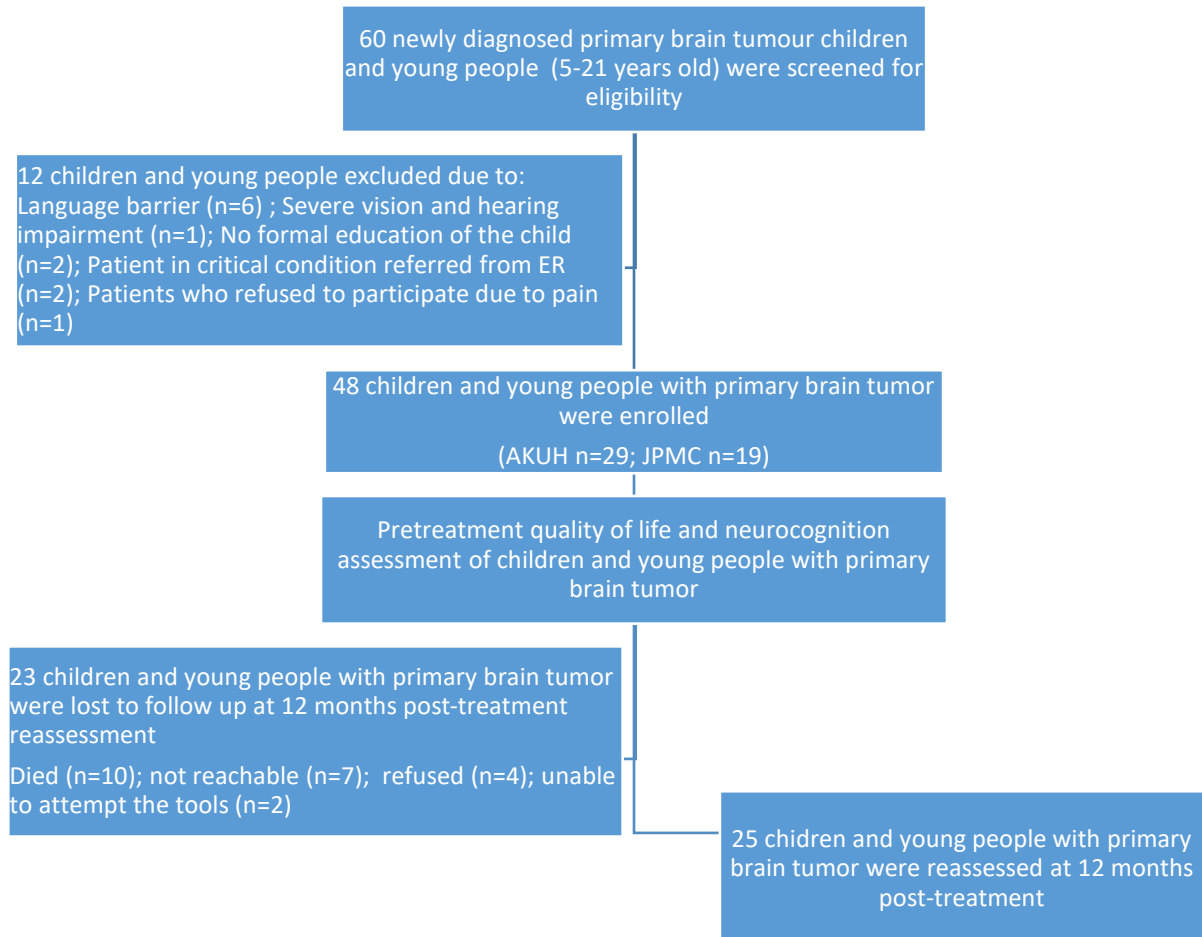

Supplement: online supplemental file 1 [file bmjpo-8-1-s001.pdf]
