# Supplementary material for: Sociodemographic and clinical predictors of quality-of-life outcome in children and young people with primary brain tumour in Karachi, Pakistan: a prospective cohort study
Source: BMJ Paediatr Open. 2024 Dec 11;8(1):e002505. doi: 10.1136/bmjpo-2024-002505 (PMC11647362; doi:10.1136/bmjpo-2024-002505)
Supplement: online supplemental file 2 [file bmjpo-8-1-s002.pdf]

Supplementary 2

Risk of Bias Assessment

Sociodemographic and birth related factors participants		
**Factors**	**Followed for 12 months (n=25)**	**Loss to follow-up (n=23)**
**Demographics Related n (%)**		
**Patient's Age**		
5-9	08 (32)	07 (30)
10-14	06 (24)	08 (35)
15-17	06 (20)	05 (22)
18-21	05 (20)	03 (13)
**Median Age of the patients in years (IQR)**	13.0 (9-17)	12.0 (8-15)
**Gender**		
Male	17 (68)	12 (52)
Female	08 (32)	11 (48)
**Province of Residence**		
Sindh	20 (80)	15 (65)
Punjab	03 (12)	05 (22)
Kyber Pakhtunkhwa	02 (08)	02 (09)
Gilgit Baltistan	00 (0)	01 (04)
**Mother Tongue**		
Sindhi	03 (12)	04 (18)
Urdu	15 (60)	07 (30)
Pushto	02 (08)	03 (13)
Punjabi	02 (08)	06 (26)
Others (Saraiki and Shina)	03 (12)	03 (13)
**Educational status of the child**		
Primary	10 (40)	14 (61)
Secondary	09 (36)	06 (26)
Higher secondary or above	06 (24)	03 (13)
**Median Years of child's formal education (IQR)**	7 (1.5-10.5)	5 (1-9)
**Household Family Members**		
≤ 6	13 (52)	08 (35)
>6	12 (48)	15 (65)
**Median Number of Household Family Member (IQR)**	06 (5-7.5)	08 (5.5-11)
**Number of Siblings**		
≤ 3	18 (72)	13 (57)
>3	07 (28)	10 (43)
**Median Number of Siblings (IQR)**	03 (2-4)	03 (2-4)
**Birth Related Factors of the patient n(%)**		
**Gestational age ****		
**Preterm**	06 (24)	01 (04)
**Term**	19 (76)	22 (96)
**Mean Gestational age in weeks (SD)**	36.7 (2.7)	37.7 (0.7)

**Birth Order**		
First	08 (32)	07 (30)
Middle	13 (52)	12 (52)
Last	04 (16)	04 (17)
**Median Birth order of the child (IQR)**	02 (1-3)	02 (1-4)
**Parental Sociodemographic n(%)**		
**Age of Mother (in year)**		
25-34	07 (28)	09 (39)
≥ 35	18 (72)	14 (61)
**Mean age in years (SD)**	38.6 (6.4)	38.9 (6.5)
**Age of Father (in years)**		
25-34	04 (16)	01 (04)
≥ 35	21 (84)	22 (96)
**Mean age in years (SD)**	42.7 (8.2)	43.5 (9.3)
**Marital status**		
Married	21 (84)	21 (91)
Others (Widower, Widow, Divorce)	04 (16)	02 (09)
**Educational status of Mother**		
No formal education	05 (20)	07 (30)
Primary	03 (12)	06 (26)
Secondary	05 (20)	03 (13)
Higher Secondary and above	12 (48)	07 (31)
**Median Years of Mothers education (IQR)**	10 (3-14)	05 (0-12)
**Education status of father**		
No formal education	03 (12)	07 (30)
Primary	00 (0)	03 (13)
Secondary	03 (12)	06 (26)
Higher Secondary and above	19 (76)	07 (31)
**Median Years of Father education (IQR)***	12 (11-16)	08 (0-12)
**Working status of the parents**		
Only Father Working	19 (76)	19 (82)
Only Mother Working	02 (08)	00 (0)
Both Father and Mother Working	01 (04)	02 (09)
Both Father and Mother Not Working	03 (12)	02 (09)
**Household Monthly income (in**		
**USD)**	04 (16)	04 (17)
≤ 60	09 (36)	14 (61)
60-160	04 (16)	03 (13)
160-320	08 (32)	02 (09)
>320		
**Median Monthly Income in USD (IQR)**	141.3 (70.6-583)	106 (159.0)

Current conversion rate of USD is 283
**significant at p value <0.05 by using Mann whitney U test*
***Gestational age; Preterm defined as delivery before 37 weeks of gestation; term defined as delivery between 37+0 to 41+6 weeks*

Tumor and Treatment related factors of participants with Brain Tumor		
**Factors**	**Completed the follow-up (n=25)**	**Loss to follow-up (n=23)**
**Tumor related n(%)**		
**Patient’s age at diagnosis**		
5-9	09 (36)	08 (35)
10-14	05 (20)	07 (30)
15-17	07 (28)	06 (26)
18-21	04 (16)	02 (09)
**Median age of patient at tumor diagnosis in years (IQR)**	13 (8.5-16.5)	12.0 (9-15)
**Site of treatment**		
Private Tertiary Care Hospital	18 (72)	11 (48)
Public Tertiary Care Hospital	07 (28)	12 (52)
**Location of Brain Tumor**		
Supratentorial	08 (32)	04 (17)
Cerebrum	08	02
Intraventricular	00	02
Infratentorial	06 (24)	11 (48)
Cerebellum	04	04
Brainstem	00	03
Fourth Ventricle	02	04
Suprasellar	07 (28)	05 (22)
Hypothalamus	02	02
Thalamus	00	02
Third ventricle	01	00
Infundibulum	02	00
Optic chiasma	02	00
Not known	00	01
Sellar	03 (12)	02 (09)
Pituitary	03	02
Multiple	01 (04)	01 (04)
**Histopathology of Tumor**		
Glioblastoma	03 (12)	01 (04)
Medulloblastoma	01 (04)	06 (26)
Ependymoma	01 (04)	02 (10)
Diffuse Astrocytoma	01 (04)	00 (0)
Craniopharyngioma	04 (16)	01 (04)
Pilocytic Astrocytoma	09 (36)	03 (13)
Pituitary Adenoma	02 (08)	03 (13)
Others [#]	02 (08)	01 (04)
Not known	02 (08)	06 (26)
**Grade of Tumor**		
Grade 1	16 (64)	06 (26)
Grade 11	02 (08)	08 (35)
Grade 111	01 (04)	01 (04)
Grade 1V	04 (16)	01 (04)
Not known	02 (08)	07 (31)
**Tumor Size (in mm^3)**		
≤9135	03 (12)	05 (22)
9136 – 35088	05 (20)	03 (13)
35089 – 90000	04 (16)	04 (17)
>90000	05 (20)	02 (09)
Not available	08 (32)	09 (39)
**Median Tumor Size (IQR)**	35742 (17413-96509)	17255 (5790-48504)

**History of Seizure**		
Yes	07 (28)	03 (13)
No	18 (72)	20 (87)
**Hydrocephalus***		
Present	08 (32)	15 (65)
Absent	17 (68)	08 (35)
**Family History of Brain Tumor**		
Yes	03 (12)	02 (09)
No	22 (88)	21 (91)
**Family History of any other Cancer**		
Yes	05 (20)	06 (26)
No	20 (80)	17 (74)
**Treatment Related n (%)**		
**Post-treatment Seizures***		
Yes	03 (12)	01 (04)
No	21 (84)	00 (00)
No information	01 (04)	22 (96)
**Type of treatment**		
Surgery only	14 (56)	12 (52)
Radiotherapy only	00 (0)	01 (04)
Combination	07 (28)	05 (22)
Surgery and Chemotherapy	02	02
Surgery and Radiotherapy	02	01
Surgery, Radiotherapy & chemotherapy	03	02
No intervention	04 (16)	05 (22)
**Type of Surgery ****		
Biopsy	00 (0)	01 (04)
Total resection	07 (28)	03 (13)
Subtotal resection	01 (04)	01 (04)
Maximum safe resection	13 (52)	12 (52)
No surgical intervention	04 (16)	06 (26)
**Presence of VPS/ EVD***		
Yes	08 (32)	14 (61)
Only EVD	02	02
Only VPS	03	05
EVD and VPS	03	07
No	17 (68)	09 (39)
**Recurrence**		
Yes	01 (04)	00 (00)
No	23 (92)	02 (09)
No information	01 (04)	21 (91)

#Histopathology for others include; anaplastic astrocytoma, optic chiasma and choroid plexus papilloma

**Significant at p value < 0.05 by using chi-square/ fisher exact test*

***Type of Surgery: total resection (100% of tumor removal); maximum safe resection (> 90% of tumor removal); subtotal resection (< 90% of tumor removal)*

EVD; External ventricular drain, VPS; ventriculoperitoneal shunt
