# Supplementary material for: Sociodemographic and clinical predictors of quality-of-life outcome in children and young people with primary brain tumour in Karachi, Pakistan: a prospective cohort study
Source: BMJ Paediatr Open. 2024 Dec 11;8(1):e002505. doi: 10.1136/bmjpo-2024-002505 (PMC11647362; doi:10.1136/bmjpo-2024-002505)
Supplement: Supplementary file 3 [file bmjpo-2024-002505.reviewer_comments.pdf]

PEER REVIEW HISTORY

BMJ Paediatrics Open publishes all reviews undertaken for accepted manuscripts. Reviewers are asked to complete a checklist review form and are provided with free text boxes to elaborate on their assessment. These free text comments are reproduced below.

ARTICLE DETAILS

**TITLE (PROVISIONAL)**	Sociodemographic and Clinical Predictors of Quality of Life Outcome in Children and Young People with Primary Brain tumour in Karachi, Pakistan; A Prospective Cohort Study
**AUTHORS**	zahid, nida; Enam, Syed; Mårtensson, Thomas; Azam, Iqbal; Mushtaq, Naureen; Moochhala, Mariya; Hassan, Aneesa; Kausar, Faiza; Bakhshi, Saqib; Rehman, Lal; Javed, Farrukh; Mughal, Muhammad; Altaf, Sadaf; Kirmani, Salman; brown, nick

VERSION 1 - REVIEW

**REVIEWER NAME**	***Piyush Gupta***
**REVIEWER AFFILIATION**	
**REVIEWER CONFLICT OF INTEREST**	
**DATE REVIEW RETURNED**	14-May-2024

**GENERAL COMMENTS**	Comments<br><br>Please see the attached manuscript for embedded edits, suggested changes, and comments.<br>Also, the present discussion is a bit haphazard and repetitive. For example, para 1 is duplication of introduction and can be completely omitted.<br><br>Rewrite the Discussions in four paragraphs (without any headings or subheadings) viz.<br>1. State the major findings of the study without repeating data.<br>2. Compare your results with those in literature.<br>3. State strength and limitations of your study and results.<br>4. Provide the impact of your findings on practice and policy.

VERSION 1 – AUTHOR RESPONSE

The Discussions should be in four paragraphs (without any headings or subheadings) viz.<br><br>1. State the major findings of the study without repeating data.<br><br>2. Compare your results with those in literature.<br><br>3. State strength and limitations of your study and results.	I have made all the suggested changes<br><br>**Section: Discussion**<br><br>**Page # 11: Line#373-377**<br><br>**Page # 12: Line#379-397**

4. Provide the impact of your findings on practice and policy.	**Page # 12: Line#404-411**<br><br>**Page # 12: Line# 399-402**

VERSION 2 – REVIEW

**REVIEWER NAME**	***Piyush Gupta***
**REVIEWER AFFILIATION**	
**REVIEWER CONFLICT OF INTEREST**	
**DATE REVIEW RETURNED**	05-Nov-2024

**GENERAL COMMENTS**	None
